# Lrp4 Modulates Extracellular Integration of Cell Signaling Pathways in Development

**DOI:** 10.1371/journal.pone.0004092

**Published:** 2008-12-31

**Authors:** Atsushi Ohazama, Eric B. Johnson, Masato S. Ota, Hong J. Choi, Thantrira Porntaveetus, Shelly Oommen, Nobuyuki Itoh, Kazuhiro Eto, Amel Gritli-Linde, Joachim Herz, Paul T. Sharpe

**Affiliations:** 1 Department of Craniofacial Development, Dental Institute, King's College London, Guy's Hospital, London, United Kingdom; 2 Department of Molecular Genetics, UT Southwestern Medical Center, Dallas, Texas, United States of America; 3 Section of Molecular Craniofacial Embryology, Graduate School, Tokyo Medical and Dental University, Tokyo, Japan; 4 Department of Genetic Biochemistry, Kyoto University Graduate School of Pharmaceutical Sciences, Kyoto, Japan; 5 Department of Oral Biochemistry, Sahlgrenska Academy at Goteborg University, Goteborg, Sweden; University of Giessen Lung Center, Germany

## Abstract

The extent to which cell signaling is integrated outside the cell is not currently appreciated. We show that a member of the low-density receptor-related protein family, Lrp4 modulates and integrates Bmp and canonical Wnt signalling during tooth morphogenesis by binding the secreted Bmp antagonist protein Wise. Mouse mutants of Lrp4 and Wise exhibit identical tooth phenotypes that include supernumerary incisors and molars, and fused molars. We propose that the Lrp4/Wise interaction acts as an extracellular integrator of epithelial-mesenchymal cell signaling. Wise, secreted from mesenchyme cells binds to BMP's and also to Lrp4 that is expressed on epithelial cells. This binding then results in the modulation of Wnt activity in the epithelial cells. Thus in this context Wise acts as an extracellular signaling molecule linking two signaling pathways. We further show that a downstream mediator of this integration is the Shh signaling pathway.

## Introduction

The integration of different cell signaling pathways is increasingly recognized as being of fundamental importance in development. Most attention has necessarily focused on the intracellular links between pathways since ligand-receptor-antagonist interactions that occur outside the cell are pathway specific. However the concurrent secretion of ligands in developmental processes suggests that pathways of extracellular integration must exist. Here we describe an integration between a secreted BMP antagonist, Wise (also known as USAG-1, Sosdc1 and Ectodin), and a negative Wnt co-receptor, Lrp4, that provides a novel method of extracellular communication between mesenchymal and epithelial cells based on the integration of Wnt and Bmp pathways. This integration occurs in the context of epithelial-mesenchymal signaling controlling processes that regulate tooth number.

The low-density lipoprotein (LDL) receptor family is a large evolutionarily conserved group of transmembrane proteins (for reviews, see [1], [2]). The LDL receptor was first identified as an endocytic receptor that transports the lipoprotein LDL into cells by receptor-mediated endocytosis. In this process, specific ligands are internalized after binding to their receptors on the cell surface from where they are moved to an intracellular vesicle (endosome) and then discharged to other compartments inside the cell. The LDL receptor mainly regulates the concentration of lipoproteins in the extracellular fluids and delivers them to cells (i.e. for uptake of cholesterol). More recent findings have shown that LDL receptor family members can also function as direct signal transducers or modulators for a broad range of cellular signalling pathways. For example, LDL receptor-related protein 1 (Lrp1) is involved in the modulation and integration of PDGF and TGFβ signals in smooth muscle cells of the vascular wall [3]–[5], Apoer2 (Lrp8) and its partner Vldlr controls brain development [6] and synaptic transmission [7], [8] through their common signalling ligand Reelin (reviewed in [2]), and Lrp5 and Lrp6 function as co-receptors in the Wnt signalling cascade [9]–[11]. Canonical Wnt/β-catenin signalling mediated by Lrp5 and Lrp6 plays a central role in mammalian bone density regulation [12]. Loss of Lrp5 function results in osteoporosis pseudoglioma syndrome that is characterized by a juvenile onset of decreased bone mass [13]. *Lrp4* (also called *Megf7*) belongs to the LDL receptor family and ENU-induced *Lrp4* null mutants die at birth with defects in formation of multiple embryonic tissues [14]. However, several other allelic mutations at the *Lrp4* locus have been reported that survive [15]–[17]. A retroviral-derived allele appears to be hypomorphic, because wild-type transcripts are present in these mutants [16]. A second allele was generated by targeted mutation by introducing a stop codon just upstream of the transmembrane domain. This allele is also assumed to be hypomorphic, since it has an identical phenotype to the retrovirally-derived alleles [15], [16].

Lrp5/6 have been shown to be able to modulate both Wnt and Bmp signalling by the direct binding of Bmp antagonists such as *Wise*, replacing binding of Wnts [18]–[21]. Similarly, *Lrp4* was shown to suppress Wnt signalling, likely by competing for LRP5/6 in the Wnt/Fz complex [15]. We have identified a domain in *Lrp4* that contains the highly conserved region where Wnts and Wise bind in Lrp5/6 and provide biochemical evidence that Wise can bind to Lrp4.

The tooth is an organ that develops as a result of sequential and reciprocal interactions between the oral epithelium and neural crest-derived mesenchyme. The first morphological sign of tooth development is thickening of the oral epithelium. The thickened epithelium progressively takes the form of “bud”, “cap” and “bell” configurations as differentiation and morphogenesis proceeds [22]. Epithelial cells and mesenchymal cells (dental papilla) differentiate into enamel-secreting ameloblasts and dentin-secreting odontoblasts, respectively. It has been established that many different signalling pathways such as Bmp, Fgf, Wnt, Shh and Tnf are involved at multiple stages of tooth development (for reviews, see [23]–[25]). A role for Lrps in any of these signalling pathways in tooth development has however not been established.

We report here that *Lrp4* is expressed in spatially restricted patterns in epithelial cells during tooth development. Changes in Bmp and Wnt signalling were observed during tooth development in both *Lrp4* and *Wise* mutants. *Lrp4* mutants display a range of tooth number abnormalities that are identical to those seen in *Wise* mutants and include fused molars and supernumerary incisors and molars. We observed upregulation of both Wnt and Bmp activities in *Lrp4* and *Wise* mutants that were accompanied by a downstream loss of Shh activity. The antagonism of BMP signaling by Wise thus does not occur in the absence of Lrp4. We propose that the ability of Wise to bind BMP's and to Lrp4 allows it to act as an extracelluar, mesenchyme to epithelial signaling protein that is capable of BMP with Wnt signaling.

## Results

### Interaction between WISE and LRP4

**Figure 1:**
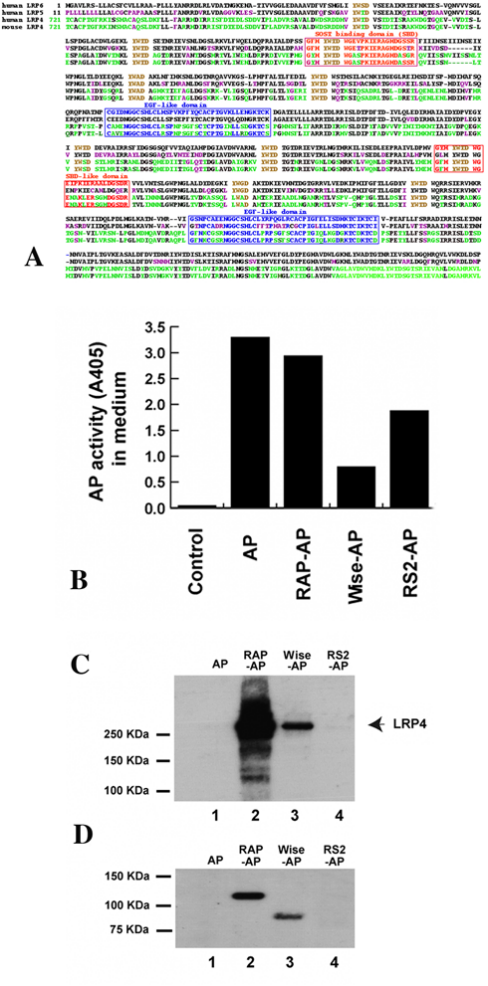
RAP and Wise, but not AP and RSpondin2 (RS2), bind to LRP4. (A) Sequence alignment of Lrp4, Lrp5 and Lrp6. Alignment of amino acid sequence of EGF-like repeats 1 and 2 of mouse in the extracellular domains of Lrp5/6 and Lrp4. (B) Media containing AP or AP-tagged proteins were produced by transfection of HEK293A cells with indicated constructs for 48 hrs. AP activity measured in media shows various expression levels and no presence of AP in control medium. (C) HEK293A cells expressing LRP4 were incubated in equal volumes of media containing indicated proteins, treated with a cross-linker dithiobis[succinimidylpropionate], and lysed prior to analysis of LRP4-binding proteins by immunoprecipitation with anti-AP antibody followed by immunoblotting with anti-LRP4 antibody. (D) LRP4 ectodomain fused with human Fc was produced as a secreted protein, conjugated to Protein A-Agarose, and incubated in equal volumes of media containing indicated proteins prior to analysis of LRP4 binding proteins by immunoblotting with anti-AP antibody.

Wise acts to modulate both BMP and Wnt signalling during development. The action of Wise on BMP signaling is as a secreted antagonist that binds BMP ligands [19], [20]. The Wnt modulation by Wise is mediated by its binding to the extracellular domain of Lrp6. [18]. The extracellular domain of Lrp6 contains four EGF repeats and Wise (a cysteine knot protein) shares repeats 1–2 of the domain of Lrp6 essential for interaction with Wnts. Alignment of the amino acid sequences of EGF-like repeats 1 and 2 of Lrp5 and Lrp6 showed this region to be highly conserved in Lrp4 (Figure 1A), raising the possibility that Lrp4 might also interact with Wise and thereby mediate the integration of Wnt and Bmp signals during morphogenesis. A similar mechanism in which Lrp1 was shown to integrate PDGF and TGFβ signals in the vascular wall has been proposed by Boucher et al. [3]. In order to test for a physical interaction between Wise and Lrp4, we performed two types of binding assays in cultured cells and *in vitro*. In the first assay, HEK293A cells expressing Lrp4 were incubated with media containing the alkaline phosphatase (AP)-tagged known ligand, (RAP), and the putative ligands for Lrp4, Wise and R-spondin2. AP-fusion proteins were then isolated with an antibody against AP and immunoblotted to detect co-precipitated Lrp4 (Figure 1C). In another assay, the converse experiment was performed were Fc-Lrp4 fusion protein was immobilized on ProteinA beads and incubated with the AP-tagged putative ligands. Bound proteins were detected by immunoblotting (Figure 1D). Both assays gave equivalent results, revealing interaction of Lrp4 with Wise, but not with R-spondin2, another modulator of the Wnt signalling pathway [26], [27]. AP and AP-RAP served as negative and positive controls, respectively.

### Expression of *Lrp4* and *Wise* in early tooth development

**Figure 2:**
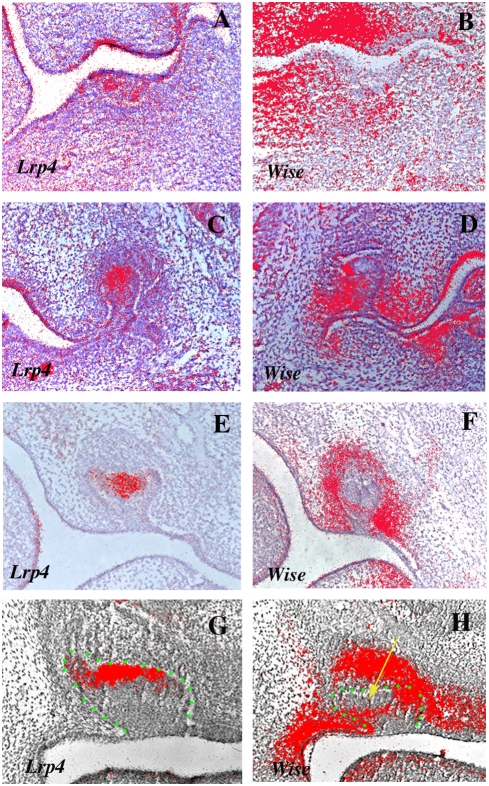
The expression patterns of *Lrp4* and *Wise* during early molar and incisor tooth development. (A, B) *Lrp4* was expressed in tooth epithelium whereas *Wise* expression was observed in tooth mesenchyme. (C, E) The expression of *Lrp4* was restricted to the primary enamel knots. (D, F) *Wise* expression was found in both epithelium and mesenchyme but was absent from primary enamel knots. (G, H) Sections showing complementary expression of *Lrp4* (G) and *Wise* (H) in incisor regions. Yellow arrow representing the region of *Lrp4* expression (H). Tooth epithelium outlined in green (G, H). Radioactive *in situ* hybridisation on frontal sections showing *Lrp4* expression (A, C, E, G) and *Wise* expression (B, D, F, H) in embryo heads at E12.5 (A B), E13.5 (C, D) and E14.5 (E–H).

In order to determine the temporal-spatial relationships between Wise and Lrp4, expression of *Lrp4* and *Wise* were analyzed in the developing heads of mouse embryos between days 12.5 and 14.5 of gestation (E12.5–E14.5) using radioactive *in situ* hybridisation. Thickening of the oral epithelium to form dental placodes takes place from E12.5. At this stage, weak *Wise* expression was observed in molar tooth mesenchyme whereas *Lrp4* was exclusively expressed in tooth epithelium (Figures 2A and 2B). At E13.5, the bud stage, the expression of *Lrp4* became restricted to the epithelial cells at tips of molar tooth buds. *Wise* expression was observed in epithelium and mesenchyme but it was absent from the tip of tooth bud epithelium (Figures 2C and 2D). At E14.5, the cap stage, *Lrp4* showed restricted expression in the primary enamel knots (Figure 2E). *Wise* expression was observed in tooth mesenchyme and thus *Lrp4* and *Wise* are expressed in a complementary manner in bud and cap stage molar tooth germs (Figure 2F). Similar complementary pattern of expression of *Lrp4* and *Wise* were observed in incisor tooth germs (Figures 2G and 2H).

### Incisor teeth in Lrp4 and Wise deficient mice

**Figure 3:**
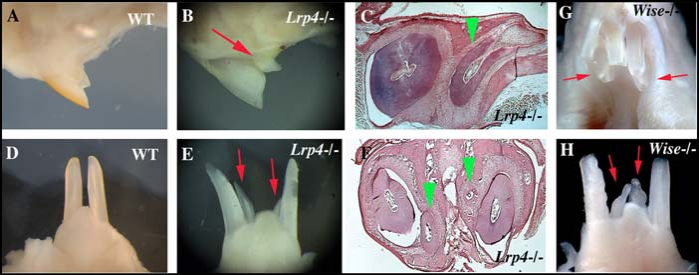
Supernumerary teeth in incisor region. Supernumerary teeth were observed in incisor region on both maxilla (arrows in B, G and arrowheads in C) and mandible (arrows in E, H and arrowheads in F) of both *Lrp4* and *Wise* mutants. Sagittal view of maxillary incisor region (A and B), lingual view of mandibular incisors (D, E and H), occlusal view of maxillary incisors (G), sagittal sections (C) and frontal sections (F) of wild-type (A, D), *Lrp4* mutants (B, C, E, F) and *Wise* mutants (G, H) at adult.

Mice have only one incisor in each jaw quadrant. Supernumerary incisors were observed in both the maxilla and mandible in *Lrp4* mutants. In the maxillary incisor region, supernumerary teeth were located on the lingual sides of each endogenuous incisor, although the locations of the supernumerary teeth were slightly variable (Figures 3A–3C). In the mandible incisor region, two supernumerary incisors were found midline between the endogenous incisors in *Lrp4* mutants (Figures 3D–3F). The supernumerary teeth had single roots and lance-like tips. All supernumerary teeth however were reduced in size and had abnormal shapes. Supernumerary incisors were also observed in both the maxilla and mandible of *Wise* mutant mice (Figures 3G and 3H; [29]). The size, shape and location of these supernumerary incisors appeared identical to those in *Lrp4* mutant mice.

### Molar teeth in *Lrp4* and *Wise* deficient mice

**Figure 4:**
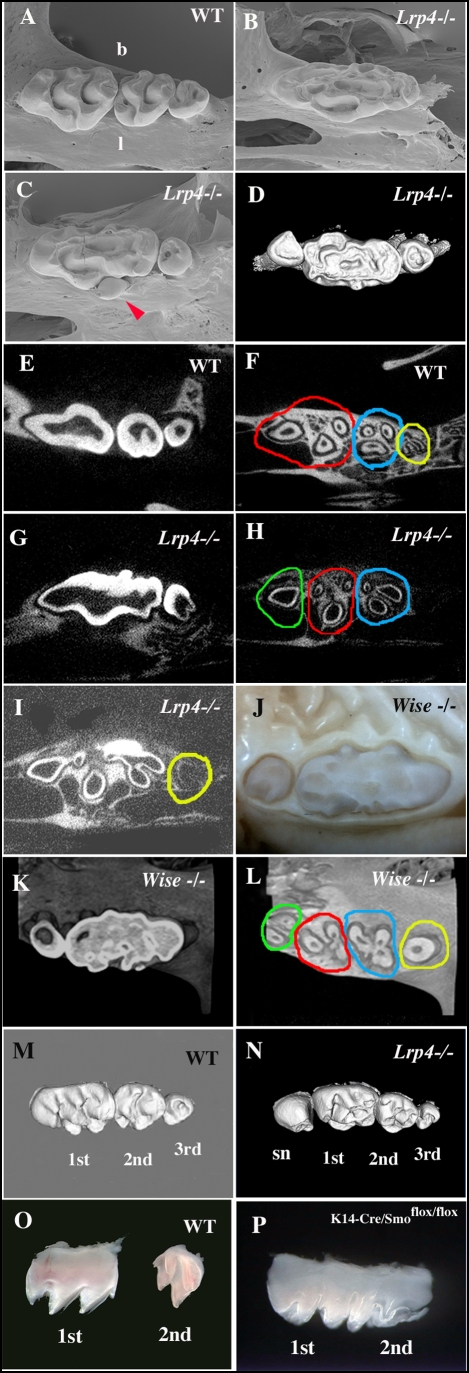
The molar tooth phenotypes of *Lrp4* mutant mice and *Wise* mutant mice. SEM images of maxillary molars (A–C), 3D reconstructions based of micro CT scans (D, M and N), horizontal micro CT sections (E–I, K and L) and dissected maxillary molars (O and P) of adult wild-type (A, E, F, M and O), *Lrp4* mutants (B–D, G–I and N) adults, *Wise* mutants (J–L) and K14-Cre/Smo^flox/flox^ mice (P) of adult (A–N) and P6 (O, P). One fused molar (B), fused molar with one relatively normal size tooth and lingual peg-shaped extra tooth (arrowhead in C), and fused molar with two reratively normal size teeth (D). Horizontal micro CT sections at the crown region (E and G) and root region (F, H and I). (F) In wild type, there are three groups of tooth roots in maxillary molars (red circle = three roots of the first molar; blue circle = three roots of the second molar; yellow circle = two roots of the third molar). (G–I) In *Lrp4* mutants, the fused molar with one normal size tooth showed four groups of tooth roots, indicating that a supernumerary tooth was present in the quadrant. The fused molars (H) had three groups of roots (one root in green circle; three roots in red circle; three roots in blue circle in H), and one normal size molar had one root as one unit (yellow circle in I), suggesting that the fused molar includes the supernumerary tooth. At the horizontal section level showing the tooth roots of the fused molars, the roots of a normal size molar could not be seen (H). Fused molars were also found in the maxillae of *Wise* mutant mice (J). *Wise* mutant fused molars also showed the several groups of roots (K and L). Supernumerary teeth were observed anterior to the first maxillary molar tooth (sn in N) of *Lrp4* mutant mice (N). Fused maxillary molar of K14-Cre/Smo^flox/flox^ mice (P). b; buccal side, l: lingual side. In all images, left side is anterior side. Scale bar = 1 mm.

Mice have only one incisor and three molars in each jaw quadrant that are divided by a tooth-less region, the diastema. In a quadrant, the first molar is the most anterior and largest molar followed progressively by the second and third molars (Figure 4A). We examined the molars of eleven *Lrp4* mutants (44 quadrants) and found none to have a normal phenotype in the maxilla. In the maxillae, 18 quadrants (out of 22 quadrants) had abnormally large teeth in the first molar position (Figures 4B–4D and S1). The occurence of second and third molars and also the presence of supernumerary teeth, anterior (mesial) to the first molars were also observed with varying degrees of penetrance (Figure S1). The remaining 4 quadrants that did not show the large teeth and a supernumerary tooth mesial to the first molar (Figure 4N and S1). In the mandible, the molar tooth phenotype penetrance was low, with only three quadrants showing a large molar and three quadrants showing supernumerary teeth (Figure S1).

To examine whether the abnormally large molars developed from a single tooth germ or were created by fusion of several molar tooth germs, micro CT analysis was performed. In wild-type jaws, each maxillary molar has several roots; three roots in the first molar; three roots in the second molar; one or two in the third molar (Figures 4E and 4F). The large maxillary molars in *Lrp4* mutants typically had seven roots that could be distinguished as being organized as three or four separate groups. In most cases, the most anterior aspect of the tooth had one root, followed by two groups of three roots each (Figures 4G–4I). Micro CT analysis of the supernumerary teeth found in the quadrants without the large molars showed these all had a single root. This suggests therefore that the large molars have formed from a fusion of first and second molars with a anterior supernumerary tooth. Other examples where quadrants had a large molar and a separate anterior supernumerary tooth indicated that this large molar root pattern was derived from a fusion of first and second molars or first, second and third molars (Figure S1).

Having shown the binding of Wise to Lrp4 and their complementary expression patterns during tooth development, we next compared the molar tooth phenotype of *Lrp4* mutants with that of *Wise* mutants. *Wise* mutant maxillary molars were also very large and had similar root patterns to *Lrp4* mutants, suggesting that their large molars were formed by a similar fusion process (Figures 4J–4L). The incidence of large molars and supernumerary teeth on the mandible of *Wise* mutants was higher than in *Lrp4* (Figure S1). In addition to fused and supernumerary molars, small lingual peg-shaped extra teeth were also evident in some *Lrp4* mutant quadrants (Figurs 4C and S1). These have also been reported to occur with low frequency in *Wise* mutants (Figure S1; [28]).

### Molecular analysis of supernumerary incisor development

**Figure 5:**
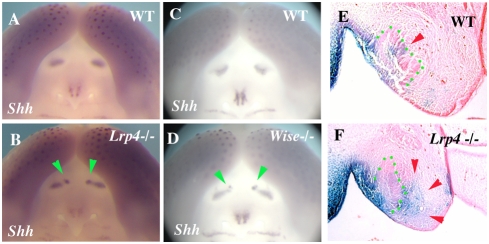
Gene expression in incisor region. (A–D) Ectopic *Shh* expression in incisor region of *Lrp4* mutant (arrowheads in B) and *Wise* mutants (arrowheads in C). (E, F) Wnt activity in enamel knot in wild-type mice (arrowhead in E). Ectopic Wnt activity in lingual side of endogeneous incisor tooth germ (arrowheads in F). Tooth epithelium outlined in green (E, F). Whole mount (A–D) and β-gal activity (E, F) showing *Shh* (A–D) expression and Wnt activity (E, F) at E14.5 in wild-type (A, C, E), *Lrp4* mutants (B, F) and *Wise* mutants (D).

Ectopic *Shh* expression was observed at incisor region of both *Lrp4* mutant at E14.5 (Figure 5B). Ectopic *Shh* expression was also observed in *Wise* mutant mice at E14.5 and its location and size were identical to it in *Lrp4* mutant (Figure 5D). It is established that both *Lrp4* and *Wise* are involved in Wnt signaling [15], [18]. In order to identify changes in canonical Wnt signalling in *Lrp4* mutant embryos, Wnt activity was detected by crossing the *Lrp4* mutant mice with BAT-gal reporter mice that express the LacZ under β-catenin/Tcf responsive elements [32]. In wild-type embryos, Wnt activity was observed in the enamel knots of endogenous incisors (Figure 5E). In *Lrp4* mutants, ectopic Wnt activity was found in lingual mesenchyme as well as the enamel knots (Figure 5F).

### Molecular analysis of supernumerary molar development

**Figure 6:**
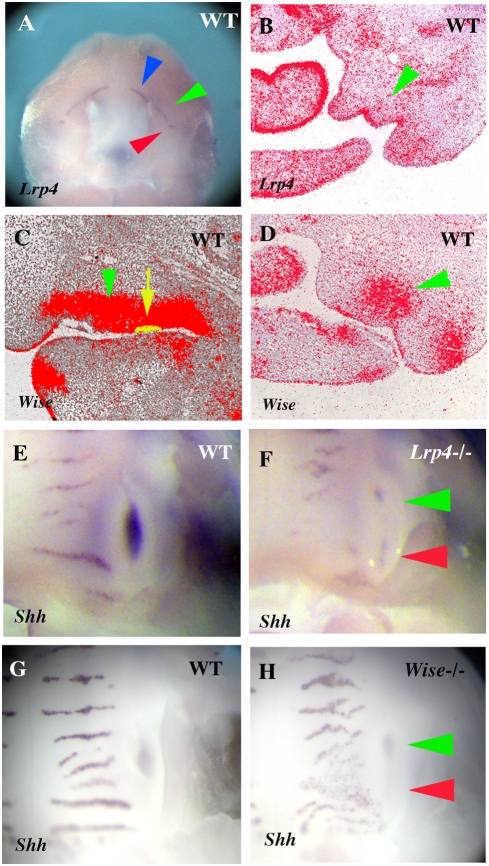
Gene expression in diastema and molar region. (A) *Lrp4* expression in incisor teeth (blue arrowhead), in molar teeth (red arrowhead) and in the diastema (green arrowhead). (B) Sections of the diastema region showed *Lrp4* expression in epithelium (arrowhead). (C, D) *Wise* expression in mesenchyme of diastema region (green arrowheads in C and D). Tooth epithelium was comfirmed by Shh expression in adjacent specimen (yellow domain pointed by arrow in C). (E–H) Ectopic *Shh* expression in diastema (green arrowhead in F and H). Reduced intensity of *Shh* expression in molar tooth germ (red arrowhead in F and H). Whole mount (A, E–H) and radioactive *in situ* hybridization (B–D) showing *Lrp4* (A, B), *Wise* (C, D) and *Shh* (E–H) expression at E12.5 (A–D) and E14.5 (E–H) in wild-type (A–E and G), *Lrp4* mutants (F) and *Wise* mutants (H). Sagittal section (C) and frontal sections (B and D).

**Figure 7:**
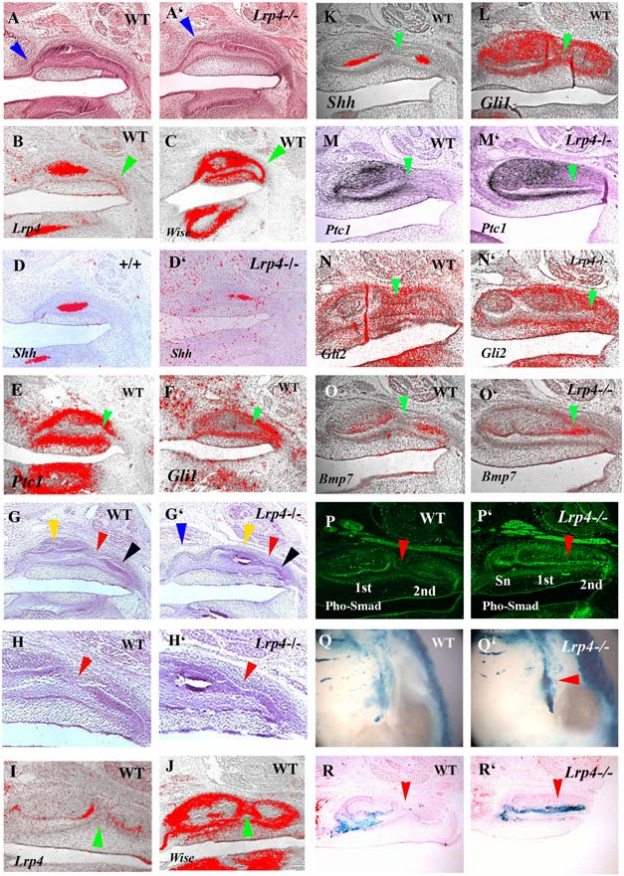
Shh, Bmp and Wnt signalling in fused maxillary molars. Sagittal section showing tooth bud epithelium of a supernumerary tooth in *Lrp4* mutant at E14.5 (arrowhead in A') and E16.5 (Supernumerary tooth; blue arrowhead, the first molar tooth germ; yellow arrowhead, the second molar tooth germ; black arrowhead in G'). Transient epithelial swelling of vestigial remants of diastema tooth in wild-type at E14.5 (arrowhead in A) and no swelling at E16 (G). Weak expression of *Lrp4* (B) and strong *Wise* (C) were observed at the posterior part of tooth germ at E14.5 (arrowheads in B, C). *Shh* expression domain was reduced in *Lrp4* mutants (D'). *Ptc1* (E) and *Gli1* (F) expression are found at posterior part of tooth germ at E14.5 (arrowheads in E, F). The first molar epithelium is still continuous with the second molar epithelium at E16.5 in wild-type (G). The junction region between the first and second molar could be distinguished by the absence of inner enamel epithelium in wild-type whereas *Lrp4* mutant showed continuouse inner enamel epithelium from first molar to second molar (red arrowhead in G–H'). H and H' are high magnification of the junction region in G and G'. Strong *Wise* expression was observed at the joint region between first and second molars but no *Lrp4* expression in the region (arrowheads in I and J). *Shh* expression are observed in only inner enamel epithelium of the first molar and second molar (K). Gli1 is expressed in junctional epithelium as well as tooth germs (L). *Gli2* and *Ptc1* expression were not observed in the epithelium of the junction region in *Lrp4* mutnats whereas they were expressed in the epithelium of the region (M–N'). *Bmp7* were upregulated at the junction region in *Lrp4* mutants whereas they were not expressed at the region in wild-type (O, O'). Phosphorylated-Smad1/5/8 (Pho-Smad) was not detected at the junction region in wild-type whereas it was found in region corresponding the junction region in *Lrp4* mutant (arrowhead in P and P'). Wnt activity was observed indistinctly in molar region of wild type (Q) whereas it was obvious in molar region of *Lrp4* mutant (Q'). Ectopic Wnt activity was also found where supernumerary molar develop (arrowhead in Q'). In wild types, sagittal sections showed Wnt activity were detected in inner enamel epithelium including enamel knot and stratum reticulum whereas it was not observed in the junction region (arrowhead in R). In *Lrp4* mutants, Wnt was activated in the junction region (arrowhead in R'). Maxillary molar tooth at E14.5 (A–F) and E16.5 (G–R') of wild-type (A, B–D, E–G, H, I–M, N, O, P, Q, R) and *Lrp4* mutant (A', D', G', H' M', N', O', P', Q' R'). Histology (A, A', G–H'), immunohistochemistry (P and P'), β-gal activity (Q–R') and radioactive *in situ* hybridisation (B–F, I–Q') on oral view (Q and Q') and sagittal sections (A–P', R, R').

Both *Lrp4* and *Wise* mutants showed supernumerary teeth in the diastema mesial to the first molars. To explore the role of *Lrp4* and *Wise* in the diastema, we examined *Lrp4* and *Wise* expression by *in situ* hybridization in this area. In the diastema region of E12.5 embryos, *Lrp4* was expressed in the epithelium, whereas *Wise* expression was observed in the mesenchyme (Figures 6A–6D). Vestigial remants of diastemal teeth can be seen in early mouse embryos as transient epithelial swellings at E13 that express *Shh* and which are rapidly eliminated at E14 by apoptosis (Figure 7A; [30], [31]). Supernumerary tooth buds that develop anterior to the first molar were visible at E14.5 in *Lrp4* mutants that were continuous with the first molar epithelium (Figure 7A'). Ectopic *Shh* expression was observed in the maxillary diastema of *Lrp4* deficient mice (Figure 6E and 6F), that was associated with retention of a vestigial swelling (blue arrowheads in Figures 7A' and 7G'). Significantly, although *Shh* was ectopically expressed, the level of expression in the developing molars was reduced (Figures 6F and 7D'). Ectopic *Shh* expression in the diastema region and reduced expresseion level of *Shh* were also observed in *Wise* mutants (Figure 6G and 6H; [28]). The retention of diastema buds mesial to the first molar in *Lrp4* and *Wise* deficient embryos thus correlates with ectopic expression of Shh.

### Mechanisms of molar fusion

The fusion phenotype (between the supernumerary tooth and first molar, and first molar and second molar) observed in *Lrp4* and *Wise* mutants suggests these molecules interact to regulate the separation of individual teeth. In order to examine the role of *Lrp4* and *Wise* in molar development, we analyzed gene expression in anterior or posterior parts of the first molar tooth germ and the anterior parts of second molar tooth germs. At E14.5, unlike in primary enamel knots, only weak expression of *Lrp4* was found in both anterior and posterior parts of the first molar epithelium (Figure 7A and 7B). *Wise* was expressed throughout the mesenchyme of these regions (Figure 7C). The expression of *Shh* was considerably reduced in the first molar epithelium of *Lrp4* and *Wise* mutants at this stage (Figure 6F, 6H and 7D') suggesting that loss of Shh signaling is linked to the molar fusion process.

The Shh receptor, *Ptc1*, was expressed weakly in posterior regions of first molar epithelium whereas strong expression was found in the mesenchyme at E14.5 (Figure 7E and S2A). The Shh signalling activator, Smoothened (Smo) and transcriptional effector *Gli1* and *Gli3* were expressed in posterior cells of first molar epithelium at E14.5 (Figure 7F, S2B and S2C). This implies that the Shh pathway is active in cells in posterior regions of the first molar epithelium at E14 although Shh is not transcribed in these cells.

In order to investigate if loss of Shh is sufficient to cause molar fusion, we examined the molars of mice with conditional mutation of Smo under keratin (K) 14 promotor (K14-Cre/Smo^flox/flox^ ; [33]). We observed fusion between first and second molars in K14-Cre/Smo^flox/flox^ mice (Figure 4P).

In wild-type embryos, second molars develop from posterior regions of first molars and then start to separate from first molar after cervical loops form which takes place at around E16.5. Micro CT scanning revealed that the fused molars contain a single, continuous large pulp chamber indicating that fusion took place before the formation of cervical loops (Figure 4G). The junction between the developing first and second molars is distinguished by the absence of a differentiated inner enamel epithelium at E16.5 (Figures 7G and 7H). In the developing molars of *Lrp4* mutants a differentiated inner enamel epithelium was present at the junction between the first and second molars (Figures 7G' and 7H'). Similar differentiated inner enamel epithelium was also observed in the junctional region in *Wise* and K14-Cre/Smo^flox/flox^ mice (Figures S3A, S3B, S4A and S4B). *Lrp4* expression could not be detected in this junctional region in wild type embryos, but *Wise* was expressed in both the epithelium and surrounding mesenchyme (Figures 7I and 7J). At E16.5, *Shh* was expressed only in the inner enamel epithelium whereas *Ptc1*, *Gli1*, *Gli2* and *Smo* were expressed in the epithelium of the junctional region between first and second molars (Figure 7K–7M, 7N and S2D). Thus, Shh signaling is active in epithelial cells in the junctional region at E16.5 with Shh being produced from the adjacent epithelium. In *Lrp4* mutants, *Ptc1* and *Gli2* were found to be slightly downregulated in the epithelium of junctional region (Figures 7M' and 7N'), suggesting that Shh signalling is reduced specifically in this epithelium.

Changes in *Bmp* expression have previously been reported in the limb buds of *Lrp4* mutant mice identifying a possible role of Lrp4 in the control of Bmp signalling [15]. The binding of the Bmp antagonist Wise to Lrp4 provides a mechanism to explain changes in Bmp signaling in *Lrp4* muatnts [19], [20]. Our identification of apparently identical tooth phenotypes in *Lrp4* and *Wise* mutants supports the premise that *Lrp4* has a role in the modulation of Bmp signalling. To examine whether Bmp signalling was altered during tooth development in *Lrp4* mutants, we performed immunohistochemistry using anti-phosphorylated Smad1/Smad5/Smad8 (phospho-Smad1/5/8) antibody. Smad1, Smad5 and Smad8 are phosphorylated by Bmp receptors following binding [34]. In wild-type molar tooth germs, phospho-Smad1/5/8 positive cells were restricted to the inner enamel epithelium and were absent from the junction regions between the first and second molars (Figure 7P). At E16.5 *Bmp4* and *Bmp7* expression could not be detected in either epithelium or mesenchyme of the junctional region but they were expressed in mesenchyme facing the inner enamel epithelium in wild-type (Figures 7O and S2E). In *Lrp4* muatnts, *Bmp4* and *Bmp7* expression was expanded into the region corresponding to the junctional region between first and second molars (Figures 7O' and S2F). Similality, a *Lrp4* mutant mice, continuous phospho-Smad1/5/8 positive cells were also found from anterior to posterior of the large fused tooth (Figure 7P'), suggesting that Bmp signalling was ectopically activated in the region corresponding to the junction between the first and second molars where fusion occurs in the mutants. Ectopic phospho-Smad1/5/8 positive cells were also found in the junctional region in *Wise* mutants whereas that could not be detected in the junctional region in K14-Cre/Smo^flox/flox^ mice (Figures S3C, S3D, S4C and S4D).

In order to identify changes in canonical Wnt signalling in molar region of *Lrp4* mutant embryos, Wnt activity was detected by crossing the *Lrp4* mutant mice with BAT-gal reporter mice. In wild-type embryos, Wnt activity was observed in tooth epithelium but was absent from the junction region of wild type at E16 (Figure 7R). In *Lrp4* mutants, ectopic Wnt activity was seen in the developing supernumerary (diastema) teeth at E14.5 (Figure 7Q') and also in the region corresponding the junctional epithelium of the fused molars at E16.5 (Figure 7R'). *Axin2* expression was upregulated at the junctional region in *Wise* mutants whereas significant differences in *Axin2* expression could not be detected in the junctional region between wild-type and K14-Cre/Smo^flox/flox^ mice (Figures S3E, S3F, S4E and S4F).

**Figure 8:**
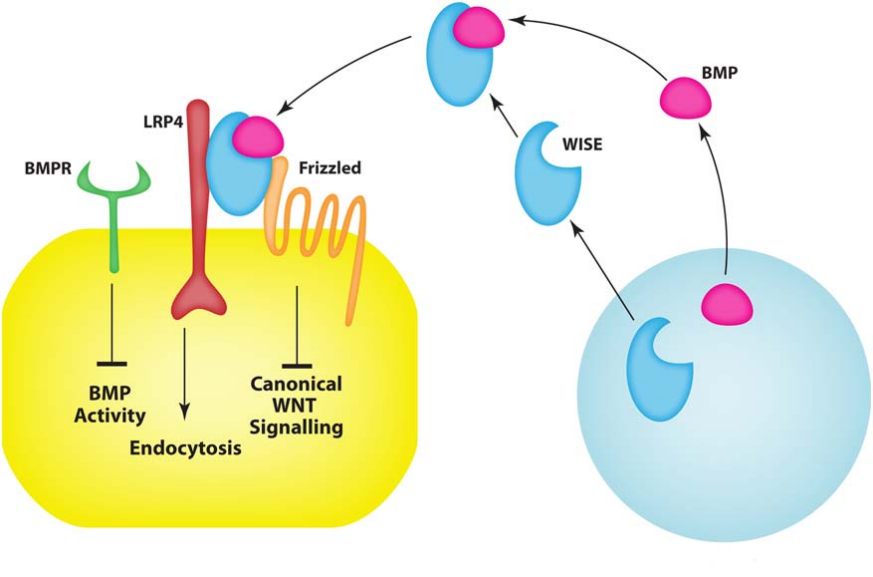
Schematic representation of *Lrp4* in tooth development. Signalling of Lrp4 and Wise regulating tooth development. BMPs bind to the Ectodin/Wise, which in turn binds to Lrp4 inhibiting Wnt signalling. In the absence of *Lrp4* or *Wise*, the excess BMPs bind to their receptor and activate both Wnt and Bmp signalling, which can result in downregulation of signalling.

Downregulation of Shh and upregulation Bmp and Wnt activity were thus present in the tooth epithelium of the junction region between the first and second molars in *Lrp4* and *Wise* mutants. These changes in Bmp and Wnt activity can be ascribed to the direct role of Lrp4 in binding Wnt proteins and the Bmp antagonist Wise. In order to determine the likely signaling hierarchy we examined Wnt and Bmp activity in the inner enamel epithelium between the developing first and second molars in K14-Cre/Smo^flox/flox^ mice. No obvious upregulation of either of these pathways was evident, suggesting that Shh lies downstream of Wnt and Bmp activity (Figure 8). The consequence of these signaling changes in the mutants is the inappropriate (ectopic) differentiation of the epithelium into inner enamel epithelium which links (fuses) the adjacent tooth germs.

## Discussion

LDL receptor-related proteins control a wide range of cellular functions including cell migration, pericellular proteolysis, signal transduction, antigen presentation, Ca influx, transcytosis and synaptic plasticity [1], [2], [35]. Originally identified through functions as endocytic receptors in lipoprotein metabolism, a fundamental role in the control of cell signalling pathways was first revealed when the LDL receptor family members Apoer2 (Lrp8) and its partner Vldlr were found to mediate the positional signals that are conveyed by the signalling protein Reelin to migrating neurons during embryonic brain development [6]. This highly conserved signalling pathway involves the clustering-induced activation of Src family tyrosine kinases and is essential for the lamination of neuronal cortical layers. Subsequently, Lrp5 and Lrp6 (*Arrow* in *Drosophila*) were found to bind Wnt proteins and act as essential modulators of Wnt signalling. Lrp4 shares overlapping structural elements within its extracellular domain with Lrp5 and Lrp6, particularly with the region that bind Wnts and Wise. Introduction of a stop codon just upstream of the transmembrane segment of Lrp4 results in mutant animals that survive but have polysyndactyly and fusion of digits [15]. This allele of *Lrp4* is thus assumed to be hypomorphic [14]–[16]. An incisor tooth phenotype was originally reported in *Lrp4* mutant mice which led us to investigate the tooth development in more detail in these mice.

The most obvious tooth phenotype we observed in *Lrp4* mutants are greatly enlarged molar teeth. These large molars arise from fusion of different molar tooth germs during development. The incidence of the different molar fusions varies between individuals but is consistently more penetrant on the maxilla than the mandible. When we compared the molar teeth from *Lrp4* mutants with those in mutants of the Bmp/Wnt antagonist Wise we observed an almost identical range of molar fusions and supernumerary teeth. In addition to the tooth phenotype, disorganised palatal rugae were observed in both *Wise* and *Lrp4* and mutants (data not shown). The only significant difference phenotypically between the two mutants was the higher penetrance of abnormal teeth on the mandible in Wise mutants, which we attribute to the fact that the *Lrp4* mutation is hypomorphic, whereas Wise is believed to be a null. This remarkable phenocopy implies that a common genetic pathway through which *Lrp4* and *Wise* control tooth development.

Since Wise had previously been shown to bind to a specific region in the extracellular domain of Lrp5/6, we aligned this region in Lrp5/6 with Lrp4 revealing a remarkable degree of conservation. Using standard biochemical protein-protein interaction assays, we found that Wise can also bind to Lrp4. Wise can modulate both the Bmp and Wnt pathways by acting as a high affinity BMP ligand antagonist and by competing for binding to Lrp's with Wnts [18]–[21]. The complementary expression patterns of *Bmp4* and *Wise* during early stages of tooth development have been interpreted as an example of a classic “activator-inhibitor morphogenetic interactions [28]. The Bmp inhibitory action of Wise was established in kidney cells and to a lesser extent in MC3T3 cells, whereas the inhibitory activity *in vivo* in tooth development has largely been inferred [19], [20], [28], [29]. Wise was originally identified as a context-dependent activator or inhibitor of Wnt signalling in Xenopus animal cap assays [18]. In this contexts however, Wise did not appear to have any effect on Bmp signalling. We detected altered Bmp and Wnt signalling in *Lrp4* mutants using phosphorylated Smads and BAT-Gal reporter mice as indicators of active Bmp and canonical Wnt pathways, respectively. Wnt and Bmp signalling was increased in those areas that are involved with molar tooth fusion, indicating that Lrp4 is essential for normal Bmp and Wnt signalling during the physical separation of molar tooth primordia. The fact that an identical phenotype was observed in Wise mutants is consistent with Lrp4 modulating Wnt and Bmp signaling by binding and thereby sequestering, presenting or endocytosing Wise in a context-dependent manner. This also implies that Wise inhibition of Bmp's may require the presence of Lrp4. However, no obvious differences in *Lrp4* or *Wise* expression could be detected in tooth germs of *Wise* or *Lrp4* mutants, respectively (data not shown).

### Fused molars

Increased Wnt and Bmp signalling and reduced Shh signalling, as a result of loss of either *Wise* or *Lrp4*, results in fusion of molar teeth. This fusion occurs when the epithelial cells in junctional regions differentiate into inner enamel epithelial cells. The reduction in Shh signalling that accompanies the increase in Bmp/Wnt activity is functionally important since conditional loss of Shh in dental epithelium also produces similar molar tooth fusions [33], [36]. K14-Cre mediated Smo deletion produced normal inner enamel epithelium differentiation in molar teeth whereas K14-Cre mediated Shh deletion resulted in a slight disruption of inner enamel epithelium [33], [36]. Another member of the LDL receptor family, Megalin, has been reported to bind Shh in addition to Bmps [37]. Loss of Megalin results in increased Bmp signalling and reduction of Shh expression in ventral forebrain development [38].

This common morphogenetic pathway may also include limb development since *Lrp4* mutants exhibit polysyndactyly with digit fusions and molecular changes that include reduction in Shh signalling [15]. In *Lrp4* mutants the differentiation of epithelium into inner enamel epithelium is normal, except for ectopic differentiation of epithelium at the junctions between developing molars.

The formation of second and third molars in mice occurs by a process that is similar to that involved in the development of permanent tooth germs from deciduous tooth germs during human embryogenesis. The essential role of Lrp4 in this process implies that this protein may play an important but as yet unidentified role in the development of the permanent teeth in humans.

### Supernumerary teeth

The formation of supernumerary teeth (mesial) anterior to the first molars, in the position of a premolar, has been described in several different mice with mutations that affect Fgf, Eda, Bmp and Shh signalling ([28], [39], [40]; Sharpe lab unpublished). The supernumerary teeth in *Lrp4* mutants closely resemble those found in *Wise* mutants and their development can be first visualised by an ectopic patch of Shh expression in the diastema at E14.5. Interestingly at this same stage, the expression of Shh in the developing molars, located a few microns more proximally, is significantly reduced in *Lrp4* mutant embryos. This suggests parallels with the context-dependent role of Wise in Wnt signalling described in *Xenopus* where it can either activate or antagonise Wnt signalling [18]. The possible primary role of Fgf signalling in supernumerary tooth formation in *Lrp4* mutants can be excluded since no changes in Sprouty expression or Fgf signalling were observed (data not shown), suggesting that the Bmp/Shh interaction lies downstream of Fgf signalling.

### The formation of supernumerary incisors

Supernumerary incisors were observed in both the maxilla and mandible of *Lrp4* mutants that phenocopy the Wise mutants [41]. In wild-type mice, vestigial tooth germs are found in the incisor region that degenerate by apoptosis during development [42]. The supernumerary incisors in *Wise* mutants are thought to form as a result of the successive development of the rudimentary tooth germs, since apoptosis is reduced in the incisor region [41]. Ectopic *Shh* expression in developing incisor regions of *Lrp4* and *Wise* mutants is indicative of the survival of rudimentary tooth germs. Bmp and Wnt signaling were also found to be upregulated in incisor regions of *Wise* mutants and which we also observed in *Lrp4* mutants [41]. Presence of supernumerary teeth in both incisor and molar regions suggests common pathways regulating molar and incisor tooth number where Lrp4 is required for the correct modulation and integration of multiple pathways.

### Lrp4-Wise interaction – a role in extracellular signaling integration

The accepted view of cell signaling by secreted proteins is that cell-cell communication is mediated by ligands binding to specific cell surface receptors that transmit an intracellular response. Secreted antagonists may also bind to the ligands to prevent pathway activation and presumably, but not well understood, the ligands must be removed from the extracellular environment, possibly by endocytosis. This removal of ligands is of critical importance in signaling in epithelial-mesenchymal interactions in development. In tooth development for example, the same ligands are repeatedly used as epithelial signals and mesenchymal signals at different times and this can only work if the ligands are removed very rapidly and effectively.

The status of Bmp proteins in the extrcellular environment is communicated to epithelial cells expressing Lrp4, which in turn modulate intracellular Wnt activitys.

## Materials and Methods

### Immunoprecipitation

HEK293A cells were transfected with pCDNA3-AP, pCDNA3-RAP-AP, pCDNA3- Wise-AP, pCDNA3-RSpondin2-AP, or pCDNA3-DKK1-AP constructs using FuGENE 6 (Roche) in DMEM plus 0.2% BSA medium to produce media containing AP or AP-tagged proteins. After 48 hr transfection, media were collected and the production levels of AP and AP-tagged proteins in the media were determined by AP activity assay using *p*-Nitrophenyl phosphate (Calbiochem) as a substrate and western blotting with anti-AP antibody (Sigma).

To test binding of AP and AP-tagged proteins to LRP4 in cell-free system, medium containing ectodomain of LRP4 was produced by transfection of HEK293A cells with pCDNA3.1-LRP4ecto-Fc constructs in DMEM plus 0.2% BSA medium. The LRP4ecto-Fc medium was incubated with Protein A-Agarose (Sigma) at 4 C for 2 hrs to make LRP4ecto-Fc-Agarose conjugates and equal volumes of media containing AP or AP-tagged proteins were precleared with Protein A-Agarose at 4 C for 2 hrs prior to incubation with the conjugates at 4 C overnight. The agarose conjugates were washed three times with PBS, resuspended in 40 ul Leammli sample buffer, and anti-AP western blotting was performed.

To test binding of AP and AP-tagged proteins in cell system, HEK293A cells were transfected with pCDNA3.1-LRP4 constructs for 48 hrs, washed once with PBS plus 0.1%BSA, and incubated in equal volumes of media containing AP or AP-tagged proteins at 37 C for 1 hr. The cells were washed once with PBS, incubated with a cross-linker, dithiobis[succinimidylpropionate] (250 mM, Pierce), at room temperature for 30 min, harvested, washed three times with PBS, and lysed in 50 mM Tris-HCl buffer, pH 7.5 containing 150 mM NaCl, 1 mM MgCl_2_, 1 mM CaCl_2_,1% Triton X-100, and protease inhibitors (Roche). After determination of protein concentration by Lowry assay, equal protein amounts of the cell lysates were subjected to immunoprecipitation using anti-AP antibody and Protein A-Agarose. The immunoprecipitates were reduced with 5% b-mercaptoethanol and anti-LRP4 western blotting was performed. The polyclonal rabbit anti-LRP4 antibody was generated in our laboratory against C-terminal peptide CWKHERKLSSESQV.

### Production and analysis of transgenic mice

*Lrp4* mutant mice were produced as described by Johnson et al. [15]. *Wise* mutant mice were produced as described by Kassai et al. [28]. Mice with a K14-Cre/Smo^flox/flox^ mice were produced as described by Gritli-Linde et al. [33]. BAT-gal mice were produced as described by Maretto et al. [33].

Day E0 was taken to be midnight prior to finding a vaginal plug. To accurately assess the age of embryos, somite pairs were counted and the stage confirmed using morphological criteria e.g. relative sizes of maxillary and mandibular primordia, extent of nasal placode invagination, and the size of limb buds. Embryos were harvested at the appropriate time and genotyped using PCR and Southern blot analysis of genomic DNA extracted from unused embryonic or extraembryonic tissue. PCR assays and Southern blot hybridization were carried out. *Lrp4* mutant mice and wild-type mice heads from E10 to newborn were fixed in 4% paraformaldehyde (PFA), wax embedded and serially sectioned at 7 µm. Sections were split over 5–10 slides and prepared for histology or radioactive in situ hybridisation. Decalcification using 0.5 M EDTA (pH 7.6) was performed after fixation of E16 and newborn mice.

### *In situ* hybridisation

*In situ* hybridisation with [^35^S]UTP-labeled riboprobes was carried out as described previously by Wilkinson [43], with modifications.

Embryonic heads were sectioned at 8 µm and floated onto TESPA(3-aminopropyltriethoxysilane)-coated slides. The slides were pretreated with 5 mg/ml proteinase K and 0.25% (vol/vol) acetic anhydride to reduce background. Hybridisation was carried out overnight in a humidified chamber at 55°C. The slides were then washed twice at high stringency in 2× standard saline citrate (SSC), 50% formamide, 10 mM dithiothreitol (DTT) at 65°C for 20 min and treated with 40 µg/ml RNAse A for 30 min at 37°C to remove any nonspecifically bound probe. The high stringency washes (at 65°C in 2× SSC, 50% formamide, 10 mM DTT) were repeated, followed by a further wash at 65°C in 0.1× SSC, 10 mM DTT. The sections were then washed in 0.1× SSC at room temperature and dehydrated through 300 mM ammonium acetate in 70% ethanol, 95% ethanol, and absolute ethanol. The slides were air-dried and dipped in Ilford K.5 photographic emulsion. Autoradiography was performed by exposing the sections in a light-tight box at 4°C for 10–14 days. Slides were developed using Kodak D19, fixed in Kodak UNIFIX, counterstained with malachite green or hematoxylin, and mounted with DePex (BDG). For photography, in some of sections, the darkfield images were inverted, artificially stained red, and combined with the brightfield image by using Adobe Photoshop.

Whole-mount in situ hybridisation was carried out as described by Pownall et al. [44] and Dietrich et al. [45]. Briefly, explants were pretreated with proteinase K at 37°C, refixed in fresh 4% PFA and then prehybridised for 5 hours at 60°C in a hybridisation buffer including 50% formamide, 50 mg/ml heparin and 50 mg/ml yeast tRNA. The proteinase K concentration was 10 µg/ml, and the length of the proteinase K treatment was modified according to the size of the tissue. The probe was added at concentration of approximately 1 µg/ml of hybridization mix. After hybridisation, tissues were washed in high-stringency conditions and preblocked in antibody blocking solution, then incubated with preabsorbed antibody. DIG-labelled antisense and sense riboprobes were detected with alkaline phosphatase-coupled anti-DIG antibodies using NBT and BCIP as the color substrates in NMT solution. FITC-labelled antisense and sense riboprobes were detected with alkaline phosphatase-coupled anti-FITC antibodies using Fast Red (Sigma). Following visualisation of the stain, the tissues were postfixed and cleared in 50% glycerol before photography.

The radioactive or DIG antisense probes or fluorescent antisense probes were generated from mouse cDNA clones that were gifts from several laboratories: Axin2 (W. Birchmeier), *Bmp4* (B. Hogan), and *Shh* (A. McMahon).

### Micro CT analysis

Heads of *Lrp4* mutant, *Wise* mutant and wild-type mice were scanned with Explore Locus SP (GE Pre-clinical imaging) high resolution Micro-CT with a voxel dimension of 8 µm. Three-dimension reconstruction was performed by three structure analysis software, Microview (GE Pre-clinical imaging).

### Immunohistochemistry analysis

After deparaffinization of sections, sections were treated by proteinase K and then incubated with antibody to Phosphorylated-Smad 1/5/8 (Cell signaling Technology). As a negative control, normal rabbit serum or normal goat serum were used instead of primary antibody. Tyramide signal amplification system was performed (Parkin Elmer Life Science) for detecting Phosphorylated-Smad 1/5/8 or active-caspase-3. Slides were mounted with Aquamount. Pictures were taken with same exposure between control, wild-type *Wise* and *Lrp4* mutant mice.

### Wnt activity detection

Tissues were fixed in 2% paraformaldehyde and 0.2% Glutaraldehyde with 1% Na deoxycholic Acid and 10% noni P40 for 30 min at 4°C. Explants were then assayed for β-gal activity by staining with XGal staining solution overnight at 37°C.

### Scanning Electron Microscope (SEM) analysis

Both jaws were coated with gold and photographed using scanning electron microscopy.

## Supporting Information

Figure S1 Frequency of molar tooth phenotypes in Lrp4 and Wise mutant mice. Red circles, blue circles and green circles represent fused tooth, relatively normal sized molar and lingual peg-shaped extra teeth, respectively.
(0.38 MB TIF)

Figure S2 Shh and Bmp signalling in molar tooth development. Ptc1 (A), Smo (arrowhead in B) and Gli3 (arrowhead in C) expression was found at the posterior part of tooth epithelium at E14.5. (A) High magnification of posterior part of tooth germ of Figure 7E. Smo was expressed in junction region between first and second molars at E16.5 (arrowhead in D). Bmp4 were upregulated at the junction region in Lrp4 mutants whereas they were not expressed at the region in wild-type (E, F). Radioactive in situ hybridisation on sagittal sections in tooth germs of embryo heads at E14.5 (A–C) and E16.5 (D–F) of wild-type (A–E) and Lrp4 mutants (F).
(2.83 MB TIF)

Figure S3 Bmp and Wnt signalling in Wise mutants mice. Differentiated inner enamel epithelium were found at junction region in Wise mutants (A, B). Phosphorylated-Smad1/5/8 (Pho-Smad) positive cells were found in region corresponding the junction region between the first molar (1st) and the second molar (2nd) in Wise mutant (C, D). sn; supernumerary tooth. Axin2 expression were upregulated at the junctional region in Wise mutants (F). B and D are high magnification of the junction region in A and C, respectively. Histology (A, B), immunohistochemistly (C, D) and radioactive in situ hybridisation (E, F) on sagittal sections in upper molar at E16.5 of wild-type (E) and Wise mutants (A–D, F).
(3.12 MB TIF)

Figure S4 Bmp and Wnt signalling in K14-Cre/Smoflox/flox mice. Differentiated inner enamel epithelium were found at junction region in K14-Cre/Smoflox/flox mice (A, B). Phosphorylated-Smad1/5/8 (Pho-Smad) positive cells could not be detected in region corresponding the junction region between the first molar (1st) and the second molar (2nd) in K14-Cre/Smoflox/flox mice (arrow in C, D). Significant differences of Axin2 expression were not found at the junctional region between wild-type (E) and K14-Cre/Smoflox/flox mice (F). B and D are high magnification of the junction region in A and C, respectively. Histology (A, B), immunohistochemistly (C, D) and radioactive in situ hybridisation (E, F) on sagittal sections in upper molar at E15.5 (E, F), E16.5 (A–D) of wild-type (E) and K14-Cre/Smoflox/flox mice (A–D, F).
(3.23 MB TIF)
