# Correction: Lrp4 Modulates Extracellular Integration of Cell Signaling Pathways in Development

**DOI:** 10.1371/annotation/f19bff3b-227a-4159-b166-7dc3c19bec43

**Published:** 2009-01-08

**Authors:** Atsushi Ohazama, Eric B. Johnson, Masato S. Ota, Hong Y. Choi, Thantrira Porntaveetus, Shelly Oommen, Nobuyuki Itoh, Kazuhiro Eto, Amel Gritli-Linde, Joachim Herz, Paul T. Sharpe

The fourth author's name appears incorrectly. It should be: Hong Y. Choi. The correct citation should read: Ohazama A, Johnson EB, Ota MS, Choi HY, Porntaveetus T, et al. (2008) Lrp4 Modulates Extracellular Integration of Cell Signaling Pathways in Development. PLoS ONE 3(12): e4092. doi:10.1371/journal.pone.0004092

