# Supplementary figures and images for: Lrp4 Modulates Extracellular Integration of Cell Signaling Pathways in Development
Source: PLoS One. 2008 Dec 31;3(12):e4092. doi: 10.1371/journal.pone.0004092 (PMC2605561; doi:10.1371/journal.pone.0004092)

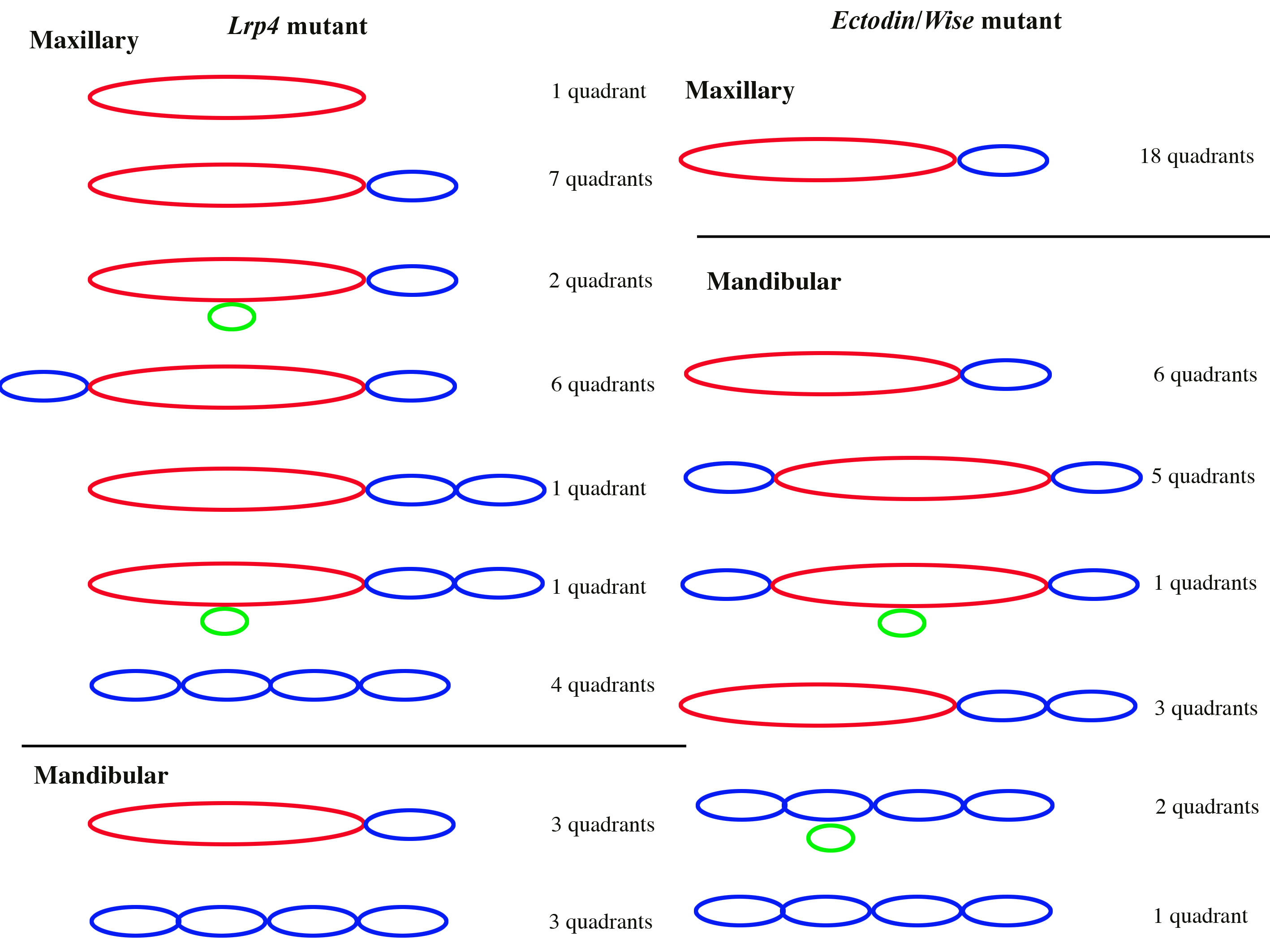

Supplement: Figure S1 — Frequency of molar tooth phenotypes in Lrp4 and Wise mutant mice. Red circles, blue circles and green circles represent fused tooth, relatively normal sized molar and lingual peg-shaped extra teeth, respectively. (0.38 MB TIF) [file pone.0004092.s001.tif]

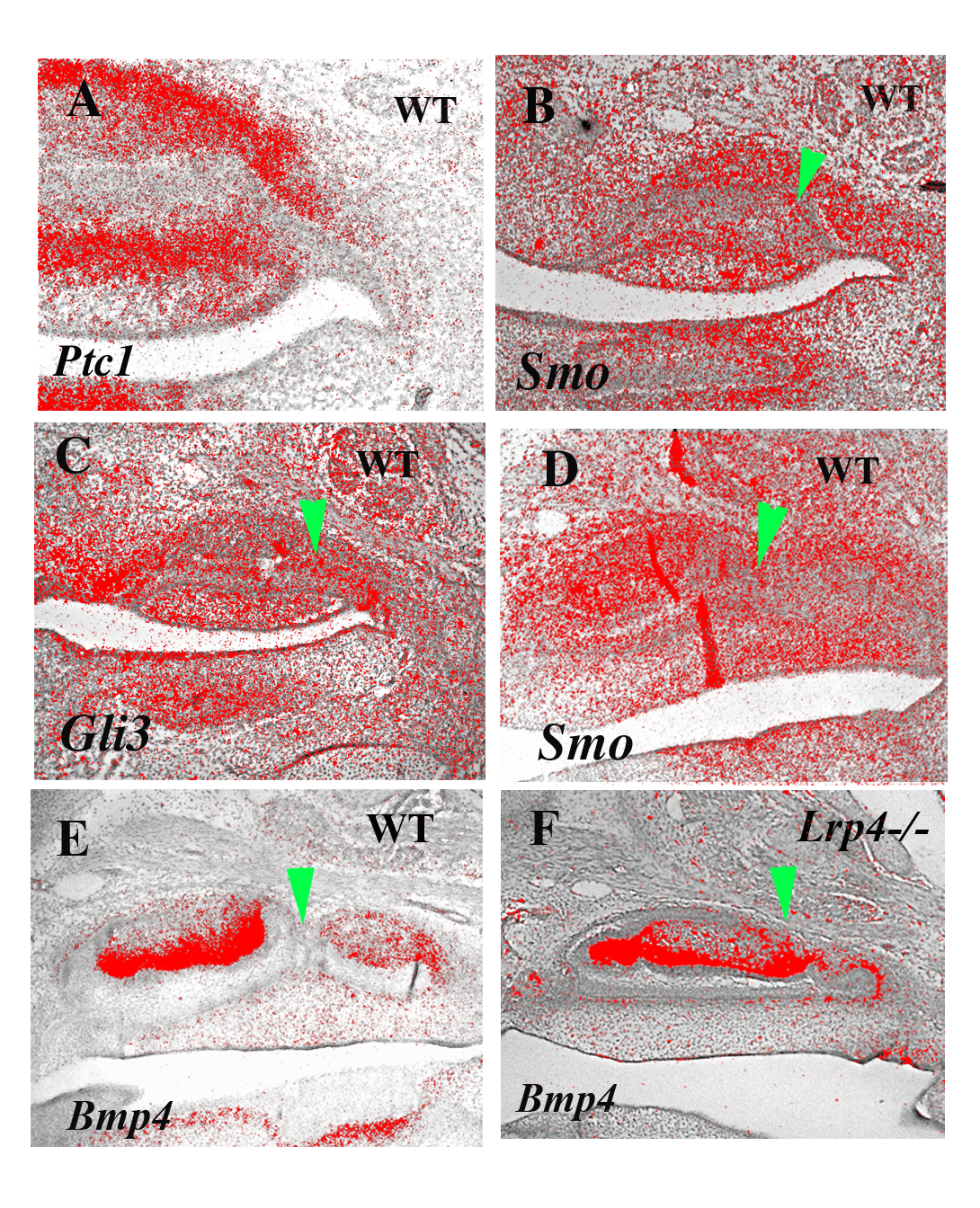

Supplement: Figure S2 — Shh and Bmp signalling in molar tooth development. Ptc1 (A), Smo (arrowhead in B) and Gli3 (arrowhead in C) expression was found at the posterior part of tooth epithelium at E14.5. (A) High magnification of posterior part of tooth germ of Figure 7E. Smo was expressed in junction region between first and second molars at E16.5 (arrowhead in D). Bmp4 were upregulated at the junction region in Lrp4 mutants whereas they were not expressed at the region in wild-type (E, F). Radioactive in situ hybridisation on sagittal sections in tooth germs of embryo heads at E14.5 (A–C) and E16.5 (D–F) of wild-type (A–E) and Lrp4 mutants (F). (2.83 MB TIF) [file pone.0004092.s002.tif]

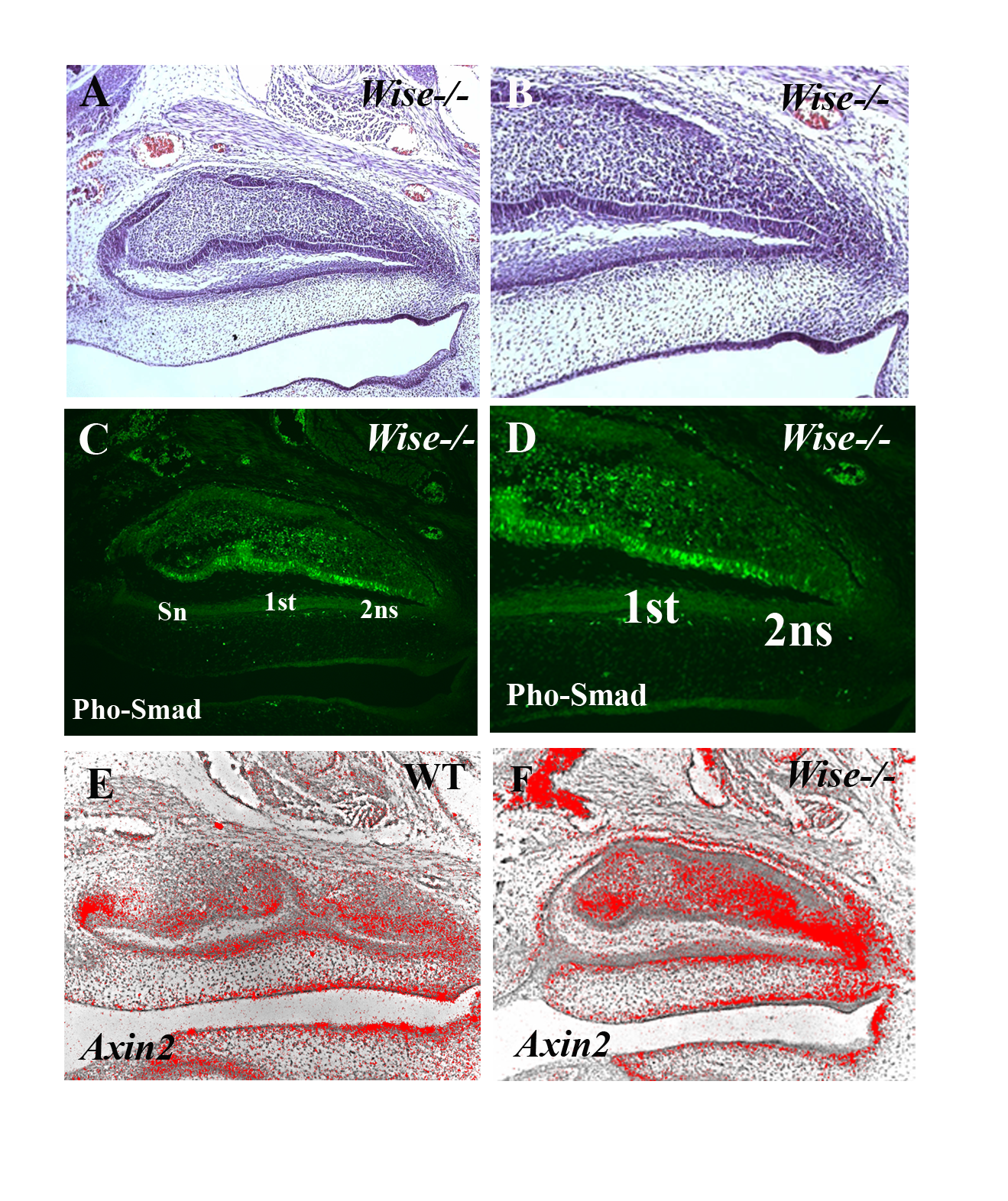

Supplement: Figure S3 — Bmp and Wnt signalling in Wise mutants mice. Differentiated inner enamel epithelium were found at junction region in Wise mutants (A, B). Phosphorylated-Smad1/5/8 (Pho-Smad) positive cells were found in region corresponding the junction region between the first molar (1st) and the second molar (2nd) in Wise mutant (C, D). sn; supernumerary tooth. Axin2 expression were upregulated at the junctional region in Wise mutants (F). B and D are high magnification of the junction region in A and C, respectively. Histology (A, B), immunohistochemistly (C, D) and radioactive in situ hybridisation (E, F) on sagittal sections in upper molar at E16.5 of wild-type (E) and Wise mutants (A–D, F). (3.12 MB TIF) [file pone.0004092.s003.tif]

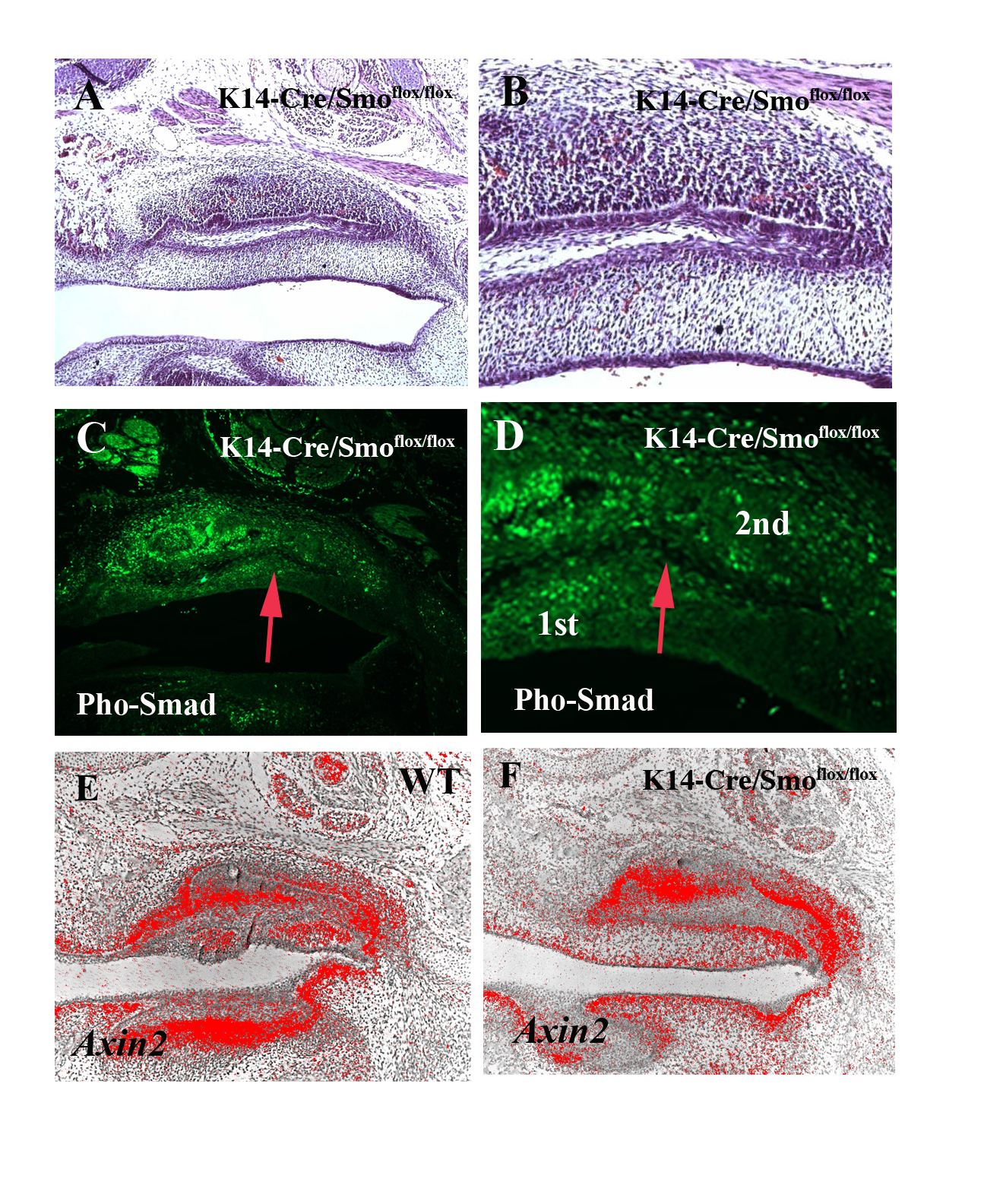

Supplement: Figure S4 — Bmp and Wnt signalling in K14-Cre/Smoflox/flox mice. Differentiated inner enamel epithelium were found at junction region in K14-Cre/Smoflox/flox mice (A, B). Phosphorylated-Smad1/5/8 (Pho-Smad) positive cells could not be detected in region corresponding the junction region between the first molar (1st) and the second molar (2nd) in K14-Cre/Smoflox/flox mice (arrow in C, D). Significant differences of Axin2 expression were not found at the junctional region between wild-type (E) and K14-Cre/Smoflox/flox mice (F). B and D are high magnification of the junction region in A and C, respectively. Histology (A, B), immunohistochemistly (C, D) and radioactive in situ hybridisation (E, F) on sagittal sections in upper molar at E15.5 (E, F), E16.5 (A–D) of wild-type (E) and K14-Cre/Smoflox/flox mice (A–D, F). (3.23 MB TIF) [file pone.0004092.s004.tif]
